# Exploring the implementation of an educational film within antenatal care to reduce the risk of cytomegalovirus infection in pregnancy: A qualitative study

**DOI:** 10.1186/s12884-024-06715-5

**Published:** 2024-08-10

**Authors:** Tushna Vandrevala, Amy Montague, Richard Boulton, Kirstie Coxon, Christine E. Jones

**Affiliations:** 1 Centre for Applied Health and Social Care Research, Faculty of Health, Science, Social Care and Education, Kingston University, London, UK; 2 Department of Psychology, Faculty of Business and Social Sciences, Kingston University, London, UK; 3 Centre for Allied Health, St George’s, University of London, London, UK; 4 School of Nursing and Midwifery, University of Central Lancashire, Preston, UK; 5 Clinical and Experimental Sciences, Faculty of Medicine and Institute for Life Sciences, NIHR Southampton Clinical Research Facility and NIHR Southampton Biomedical Research Centre, University of Southamptonand, University Hospital Southampton NHS Foundation Trust , Southampton, UK

**Keywords:** Congenital Cytomegalovirus CMV, Normalisation Process Theory, Implementation science, Improvement science, Healthcare education

## Abstract

**Background:**

Congenital cytomegalovirus (CMV) infection is a leading cause of sensorineural hearing loss and neuro-disability in childhood. In the absence of a licensed vaccine, adoption of hygiene-based measures may reduce the risk of CMV infection in pregnancy, however these measures are not routinely discussed with pregnant women as part of National Health Service (NHS) antenatal care in the United Kingdom (UK).

**Methods:**

An exploratory qualitative study was conducted, underpinned by Normalization Process Theory (NPT), to investigate how an educational intervention comprising of a short film about CMV may best be implemented, sustained, and enhanced in real-world routine antenatal care settings. Video, semi-structured interviews were conducted with participants who were recruited using a purposive sample that comprised of midwives providing antenatal care from three NHS hospitals (*n* = 15) and participants from professional colleges and from organisations or charities providing, or with an interest in, antenatal education or health information in the UK (*n* = 15).

**Findings:**

Midwives were reluctant to include CMV as part of early pregnancy discussions about reducing the risk of other infections due to lack of time, knowledge and absence of guidance or policies relating to CMV in antenatal education. However, the educational intervention was perceived to be a useful tool to encourage conversations and empower women to manage risk by all stakeholders, which would overcome some identified barriers. Macro-level challenges such as screening policies and lack of official guidelines to legitimise dissemination were identified.

**Discussion:**

Successful implementation of education about CMV as part of routine NHS care in the UK will require an increase in awareness and knowledge about CMV amongst midwives. NPT revealed that ‘coherence’ and ‘cognitive participation’ between service members are vital to imbed CMV education in routine practice. ‘Collective action’ and ‘reflexive monitoring’ is required to sustain service changes.

**Supplementary Information:**

The online version contains supplementary material available at 10.1186/s12884-024-06715-5.

## Introduction

Congenital cytomegalovirus (CMV) is the most common congenital infection worldwide, with estimated 0.3–1% of babies born with CMV infection per year [1–3]. CMV infection causes no symptoms, or only mild symptoms, in healthy adults, including the pregnant individual and most infants born with congenital CMV infection do not have obvious signs or symptoms of infection. However, up to 25% of infants with congenital CMV will have long-term adverse outcomes, including hearing loss, cognitive deficits, and to a lesser extent visual impairment [2, 4, 5]. The risk of severe health consequences of congenital CMV is highest when transmission occurs within the first trimester and infected fetuses are significantly more likely to develop severe neurological sequalae, including sensorineural hearing loss, than those infected outside of the first trimester [6]. CMV transmission occurs through contact with infected bodily fluids, particularly saliva and urine of young children, who shed the virus for prolonged periods of time. Therefore, pregnant individuals who have young children are at an increased risk of infection because of the higher rate of contact with infected saliva and urine [7]. There is currently no vaccine available outside of clinical trials for the prevention of CMV infection, and in the UK, there is no universal antenatal screening in pregnancy or for newborn infants. Therefore, CMV infection in pregnancy or the neonatal period is often not identified, meaning the opportunity for anti-viral treatment is missed. Adoption of hygiene-based measures to reduce the risk of acquisition of CMV infection in pregnancy is the only currently available preventative strategy [8, 9].

Despite the prevalence of congenital CMV infection and the significant impact on the individual child and their family, there is little awareness of CMV amongst UK pregnant women or the broader community [10–14]. When given the opportunity, pregnant women are receptive to health messages about CMV [11, 12] however, there is limited education provision embedded in antenatal care about CMV infection and risk reduction measures [15]. Pre-conception adoption of risk reduction measures are important to decrease the number of cases of CMV infection early in the first trimester of pregnancy, when severe congenital infection occurs and these messages should be reinforced – or introduced – at the first antenatal appointment for all women. Inclusion of information about CMV in pre-conception counselling or during antenatal visits should help to normalise knowledge of CMV in the population and may reduce the risk of CMV acquisition not only in the current pregnancy, but also in subsequent pregnancies. However, these measures cannot prevent all cases of congenital CMV, as in utero transmission occurs not only from primary CMV infection and infection with different strains of the virus, but also from reactivation of latent CMV [16]. Therefore, vaccine strategies remain crucial to combatting congenital CMV.

Broadly, research has described the current information about infectious diseases provided to pregnant women in primary care, as “insufficient”, with CMV information being especially inadequate [17–19]. When exploring this, antenatal care providers report reluctance in discussing CMV due to lack of time, confidence and concerns for the wellbeing of the pregnant woman upon learning of CMV risks [15, 20]. Likewise, there is lack of awareness and knowledge of CMV infection and risk-reduction measures amongst healthcare professionals [21–23]. Recent research in Australia reported up to 80% of maternity professionals sampled had not received education about CMV and only 10% were routinely discussing CMV with pregnant women [20, 24]. Likewise, in a UK study, 60% of midwives surveyed did not feel confident in their knowledge of CMV (Woods, 2017). The UK National Institute for Health and Care Excellence (NICE) Antenatal Care guidelines (NG201) have recently been updated with the recommendation to discuss CMV as part of antenatal education (https://www.nice.org.uk/guidance/ng201/chapter/Recommendations). Therefore, it is considered an essential part of antenatal care and vital to explore ways to meaningfully integrate these messages into routine care, without compromising the other important information about maintenance of health in pregnancy, or inadvertently raising anxiety in women.

Research highlights the benefits and effectiveness of using digital educational interventions to encourage behaviour change amongst pregnant women [25–27]. Our project team developed a short evidence-based film to educate pregnant women about CMV and ways to reduce their risk of acquiring CMV in pregnancy [11, 28]. A feasibility randomised controlled trial (RCT) showed that pregnant women in the intervention group were more knowledgeable about CMV, considered themselves personally susceptible to CMV infection and reported lower participation in activities which could increase CMV transmission, than those in the ‘treatment as usual’ group [28]. Furthermore, a process evaluation found that midwives who participated in the aforementioned RCT perceived the film to be an effective way to overcome barriers associated with CMV education and had the potential to increase their knowledge of CMV and confidence in having conversations about CMV infection with pregnant people [15]. The integration of a digital resource into routine antenatal care could therefore be an effective method to provide CMV education to women and to midwives, overcoming those barriers which cause reluctance to discuss CMV infection in pregnancy. However, currently little is known about how such an intervention could be successfully implemented and sustained in routine antenatal care.

Understanding context when it comes to implementing complex health interventions is crucial to its success [29, 30] and the use of theoretical frameworks is recommended to understand implementation processes [31]. One such framework is Normalization Process Theory (NPT). NPT focuses on the circumstances by which a new process is implemented, embedded, and integrated into routine practice. By understanding and considering these processes a new practice is more likely to become a ‘norm’, which is defined as “notions of how beliefs, behaviours, and actions should be accomplished” ([32]p.2). NPT focuses on ‘the work’ of individuals and groups in enacting a new practice. Our study takes a novel approach to NPT by identifying potential implementation challenges in advance, rather than reflecting on the process retrospectively. This is of particular importance to the implementation of a CMV short educational film because of the concerns raised by health care professionals who would be ‘enacting’ the new practice. NPT proposes four different concepts within its framework which are important to understand when trying to implement a new practice. *Coherence*, a form of ‘sense-making’, namely how individuals understand the new practice and how it compares with other current practices. *Cognitive Participation*, which refers to individuals’ drive and motivation to involve themselves and engage with a practice and thus maintain the practice. *Collective Action*, represents the impact of a new practice on the group and current group practices, and finally, *Reflexive Monitoring*, is how an individual understands a practice’s sustainability and what criteria they use to continually evaluate its usefulness and effectiveness in the short and long term. In this study, we used NPT to theorise and anticipate likely barriers and facilitators to wider implementation and ‘scale-up’ of CMV education during antenatal care ([32]p.5).

The aim of this study was to investigate how a short film about CMV infection and risk reduction measures can best be implemented in routine antenatal care.

## Methods

### Design and setting

This study used an exploratory qualitative descriptive approach [33]. Normalisation Process Theory provided the basis for the questionnaire schedule and the thematic analysis framework [34]. Interviews were carried out with midwives from three National Health Service (NHS) hospitals and fourteen organisations that provide, or have an interest in, antenatal education or health information in the United Kingdom (UK).

### Ethical approval

Ethical approval to carry out the project was granted by the *(name of university)* Research Ethics Committee (internal REF #2795). Potential participants were provided with an information sheet and asked if they would be willing to participate in a video interview to discuss their perspectives on the implementation of a digital CMV educational intervention into routine antenatal care. Informed consent was obtained from all participants and each participant received a £20 gift voucher in recognition of their time and expertise.

### Sampling and recruitment

We aimed to purposively sample midwives providing antenatal care, along with participants from fourteen professional colleges, organisations, or charities providing, or with an interest in, antenatal education or health information (organisational names for individual quotes supressed for anonymity). The involvement of a range of stakeholders was designed to facilitate a systemic understanding of the issues that may inform implementation of CMV in antenatal education in pregnancy.

Research midwives (or other antenatal healthcare professional) at each of the three hospitals identified relevant clinical members of staff and made the initial introductions to the interviewer (AM). Other participants were recruited from the fourteen professional colleges, organisations or charities. These organisations were identified by the research team or by stakeholders involved in a policy Roundtable meeting about CMV risk reduction in pregnancy held to disseminate findings of our previous work. Once initial connections had been made, snowball sampling was also employed.

### Data collection

Interviews were conducted on video conferencing software by a qualitative researcher with expertise in health psychology (AM). Interviews were conducted virtually due to measures in place during the COVID-19 pandemic and for convenience to connect with different sites and organisations across the UK. The interview guide was developed using the concepts from Normalization Process Theory (NPT; coherence, cognitive participation, collective action, reflexive monitoring) (Table 1). As part of the interview, participants were shown a short film (duration of approximately 2 ½ mins) about CMV infection and measures to reduce the risk of catching CMV in pregnancy (referred to as ‘the intervention’). This film was developed from a longer version, developed as part of the RACE-FIT study [28]. Interviews lasted 28—68 min (averaging 45 min) and were recorded, transcribed and anonymised. Transcription was either carried out using Microsoft Teams captions, which were then checked for accuracy and anonymised, or through a private transcription company who anonymised the data as part of the transcription process.

**Table 1:** Interview Guide developed using NPT principles

• Why do you think CMV is not currently included in antenatal education?
• Who should be providing CMV education, and when, where should it take place?
• Can you tell me about how discussing CMV with pregnant women might differ from discussing other antenatal problems?
• What are your perspectives of the CMV film?
• What do you think pregnant women’s perspectives of this film might be?
• What are you own personal reasons for including CMV education into your practice?
• Do you have any concerns in using the CMV film in your practice?
• What impacts may the inclusion of this film have on current practice?
• How do you think other antenatal providers will use this film?
• Do you think including CMV education/ and or the film will impact any working relationships?
• How do you think we could monitor the inclusion of CMV education/ the film?
• Do you anticipate any barriers to including this film in your practice?
• How can we evaluate the long-term success and impact of this film?

### Data analysis

Data collection and analysis proceeded concurrently. Once data was collected, it was then analysed thematically using Nvivo 14 [35]. Inductive thematic analysis was used initially to develop relevant themes from the data [36]. Following familiarisation with the data, codes were then generated around barriers and facilitators to CMV education according to participant attitudes and perspectives. Codes were agreed through discussion with second researcher (TV) to maximise the methodological and interpretive rigour of the analysis [37]. Once a list of codes had been established, the codes were reassembled into themes, which related to and made sense of the connections (comparisons) between the codes. To guide the overall analysis, we made memos and informally written observations about participants’ experiences in Nvivo which offered initial reflections on any potential relationships [38]. These memos were actively used during the analysis to elucidate the various processes and structures that emerged in the style of NPT.

Once the themes had been agreed, they were mapped on to the NPT constructs of coherence, cognitive participation, collective action, and reflexive monitoring (Table 2). Mapping the themes onto NPT theoretical constructs allowed for an overview of current routine practice in comparison to the proposed CMV intervention [39]. This illustrated where normalisation would likely need to take place in order for the intervention to be successfully implemented. NPT provided a useful framework within which to conceptualise the normalisation of the intervention. There was some overlap between the themes and NPT sub-components. But generally, the themes were consistent to NPT core constructs as the initial questionnaire was structured using NPT. An overview of the mapping process can be seen in Table 2 (full data structure in appendix 1), which illustrates the progression from participants’ quotes to theoretical concepts. The final data structure was agreed with all authors.

**Table 2:** Excerpt of theme mapping process

Themes	Sub-themes	NPT component	NPT core construct
CMV is not serious	Severity	Individual specification	Nature & attitude towards condition = Coherence
More confident to discuss CMV	Self-efficacy Autonomy & Empowerment	Enrolment	Motivation to engage with intervention = Cognitive participation
Senior staff support changes & champion issue	Time & opportunities	Skill set Workability	Opportunities and Barriers at NHS Trust level = Collective action
Endorsement from recognised national bodies	National drivers and guidance	Systematization	Systemic level Barriers and Opportunities = Reflexive monitoring

In the quotes provided, (…) indicates that material has been omitted, material in brackets [] was added for clarification by the authors and pseudonyms are used to protect the anonymity of participants involved in the study.

## Results

### Participants

A total of thirty participants took part in the interviews. Fifteen antenatal care providers, all of whom had a role in midwifery, were recruited from three NHS sites: one located in Southwest England (*n* = 5), one in London (*n* = 5), and one in Southeast England (*n* = 5). Examples of roles include research midwife, consultant midwife, and community midwife (see full list in Table 3). From fourteen organisations, a further fifteen participants were recruited. Seven participants were recruited from private or publicly-funded organisations who provide digital education resources to NHS Trusts or directly to the public e.g., Clevermed (https://www.clevermed.com/badgernet/), Wessex Healthier Together (https://what0-18.nhs.uk), Bounty (https://www.bounty.com), Best Beginning (https://www.bestbeginnings.org.uk)) and seven participants who worked within organisations with a particular focus on antenatal care or education e.g., (Royal College of Obstetricians and Gynaecologists (https://www.rcog.org.uk), Motherhood Group (https://www.themotherhoodgroup.com)) and one from a charity focussed on CMV (CMV Action (https://cmvaction.org.uk/)). Education providers were included to understand their experiences of working with healthcare providers to shape best practice.

**Table 3:** Participant Characteristics for NHS antenatal care providers (*n* = 15). All information was self-identified by participants

Age	20–29	2
	30–39	8
	40–49	2
	50–59	2
	60–69	1
Gender	Female	15
	Male	0
Ethnicity	White British	12
	Black British	1
	Carribean	1
	Mixed	1
Length of time in NHS	1–5	3
	6–10	7
	11–15	2
	16–20	1
	21–25	1
	26–30	1
Current position in NHS	Research midwife	2
	Antenatal Screening coordinator midwife	1
	Consultant midwife	1
	Clinical Midwife	5
	Quality improvement & governance midwife	2
	Community midwife	3
	Digital lead midwife	1

The majority of participants self-identified as female (*n* = 28/30; 93%) and predominantly White British (*N* = 24/30; 80%). The mean number of years clinical participants had worked in the NHS was 10.8 years (range 2.5 to 27 years) and the mean number of years for non-NHS antenatal education providers (organisations, charities, professional bodies) had worked for their organisation was 8.4 years (range 3-months to 21 years). Those working in organisations outside of the NHS were all in senior positions with the company or charity (Table 4).

**Table 4:** Participant Characteristics for non-NHS participants (organisations, charities, professional bodies) (*n* = 15). All information was self-identified by participants

Age	20–29	2
	30–39	3
	40–49	5
	50–59	4
	60–69	1
Gender	Female	13
	Male	2
Ethnicity	White British	12
	Black	1
	Mixed	2
Length of time in position	Less than a year	1
	1–5	5
	6–10	3
	11–15	2
	16–20	1
	21–25	1
	Missing	2
Current non-NHS position	Regulatory affairs manager	1
	Head of Department (communication, public relations, maternity, content)	5
	Education Course director	3
	Organisation Member	1
	Diversity research fellow lead	1
	Project manager	2
	Organisation founder	1
	IT developer	1

The themes that follow are structured around the NPT core constructs of coherence, cognitive participation, collective action, and reflexive monitoring, as related to our findings.

#### Coherence: Sense-making Individual level factors: CMV as a problem that needs solving

Midwives expressed a lack of knowledge and shared understanding about CMV, indicating a lack of coherence in relation to the intervention. Low perceived incidence and severity of CMV infection in pregnancy were also prominent issues raised by midwives. Midwives described having little exposure to and experience with CMV infection. This lack of exposure further exacerbated the perception that CMV was uncommon. Midwives felt unequipped and lacking confidence to discuss CMV and were concerned that pregnant families would perceive them as unknowledgeable. This lack of confidence was ultimately perceived to impact the midwife-pregnant women relationship. Midwives suggested that staff education would be a logical first step to successful implementation.

“(CMV) Isn’t that common, but it’s not exactly rare either. So, giving us the knowledge and the confidence to talk about it in a bit more detail, because I generally thought CMV was just to do with cat poo and that’s about it.” (P7, Midwife, 13 years experience)

“And then I guess looking stupid maybe, because you don’t have the answers and saying actually, “I don’t know”. It’s quite hard when you’re a medical professional, ‘cause you’re all supposed to know all these things.” (P5, Community Midwife, 6 years experience)

Likewise, a digital provider representative shared confusion about CMV, and whether it should be integrated into their platforms because of a lack of existing knowledge:

“Yeah here’s something that wasn’t in the app and we want to have something on it.. but at the same time, what we really want is to know where does it sit in the system?” (Private digital antenatal provider, 2 years experience)

Without an understanding of the risks CMV poses, midwives expressed reluctance about having conversations with pregnant women. They wanted clarity on the significance of CMV, mentioning the need for evidence to help emphasise the risks associated with infection, and its prevalence.

“How often do women come across it? How much is it a problem? … And you know, if 100% of women who get it have that result, then obviously it’s really important. But then, if only half a percent of women ever come across it. It changes the game a little bit.” (P4, Midwife, quality lead, 7 years experience)

These perspectives might be perceived as a professional challenge to the guidance that CMV education needs to be implemented, but most likely demonstrate a lack of knowledge of the importance of CMV infection in pregnancy and the recommendations to include risk reduction measures as part of antenatal information. With CMV education not currently being part of routine practice, midwives may be left feeling confused, leading them to question the need for specific discussions regarding CMV and why this was not already included in antenatal education.

“They probably just don’t want to learn about, if they’ve not had to all these years... Being in practice, why should they now really?” (P1 Midwife, Antenatal screening coordinator, 7 years experience)

Midwives discussed feeling overwhelmed with their workload and there were concerns for the lack of time to integrate more information or new practices. It is possible that they may also feel disgruntled that they did not have an opportunity to challenge new guidance, or simply not know about the recommendation to include information of CMV at the first antenatal appointment in the NICE guidelines (NG201). Provision of information about CMV was perceived as being ‘another thing’ they were required to do.

“I’m not saying it’s not a priority, and that’s probably what we need to be doing, but it’s probably not seen as priority in comparison to everything else that you’re trying to deliver in a short period of time.” (Research Midwife, 9 years experience)

The relevance of CMV education for all pregnant women was questioned by some interviewees. Midwives explained that although pregnant women who already had children were more likely to be at risk of CMV transmission, it is first-time pregnant women who showed greater engagement with antenatal education. Midwives suggested that women pregnant for the second – or more- time were harder to engage in antenatal education but for whom the risk reduction measures were most pertinent. This emphasises the need to include information about CMV during each pregnancy, as the information may be received in the first pregnancy and retained during subsequent pregnancies.

“They [women with subsequent pregnancies] are going to be the ones that you struggle with, particularly because, uhm… they’ve done it. They feel like they’ve done it. They don’t realize that the advice changes throughout pregnancy or throughout the years. Throughout each pregnancy. The advice tends to change and sometimes they are a bit stuck in their ways.” (P8, Community Midwife, 2.5 years)

There was concern that women in their first pregnancy might believe CMV education does not apply to them because they do not have other children and are therefore ‘low-risk’. This leads to unique challenges when it comes to antenatal care providers providing CMV education, firstly, ensuring it is disseminated coherently and engaged with by all pregnant women, but also ensuring the messages are felt to be personally relevant and applicable too.

“I’m not sure how women would react to it who aren’t having their first baby... I think it’s very much geared towards the family situation, set up with the toddler at home. Women having their first baby might be a bit like, OK, well I don’t have a snotty nosed kid at home so what do I need to do, or what else is going to put me at risk? I think those are the points at which you might, one might be adding concern and worry into a pregnancy without much sort of reassurance of what can be done.” (P11, Midwife, 7 years experience)

#### Cognitive Participation: motivation to engage with intervention

Midwives identified multiple benefits of the short educational film about CMV, with the majority suggesting the film would be advantageous to their current practice. This indicates there was a high level of cognitive participation among midwives to “do the work” of signposting to the educational film as part of early pregnancy appointments. Midwives were motivated to use the film to raise awareness of CMV, not only for women, but more widely to partners, families and society. They considered that the film was useful to educate women to discuss CMV with others and enable them to be more autonomous in risk reduction measures during their pregnancy.

“Yeah, I think you’ll find... What happens is with women they’ll discuss with their friends and then it becomes... Like those sorts of discussions.” (P5, Community Midwife, 6 years)

The film was perceived by antenatal clinicians and digital health information providers as a useful tool to help initiate conversations and answer questions about CMV infection in pregnancy, reducing time pressures during face-to-face consultations. Midwives felt that they personally gained knowledge from watching the film, and it left them feeling more comfortable in having conversations about CMV infection with pregnant women.

“it also puts the ownership on women as to where to access their information, so they’re not always just passively sat waiting for the midwife to tell them stuff.” (NHS digital antenatal provider, 7 years experience)

“that’s a really difficult conversation to have because people are very, very confused and you know. And actually if it’s on the screen, that’s much more easier to show.” (Private digital antenatal provider, 2 years experience)

Participants had several concerns relating to the welfare of pregnant women. Information about CMV infection in pregnancy was perceived as confusing or ‘overwhelming’ for pregnant women and the risk-reduction behaviour changes as anxiety provoking. This demonstrated contemplation of the work required to help women make sense of and, if needed, to manage risks related to CMV (in NPT terms displaying the need for cognitive participation between midwives and patients). CMV education might be perceived by antenatal providers to contribute to the number interventions designed to reduce risks in pregnancy, any of which may distort women’s perception of pregnancy as straightforward or cause them to perceive their bodies, or even those of their young children, as potentially risky to the unborn child. These quotes demonstrate that anticipating and managing this anxiety is considered as part of antenatal care. Some participants described wanting to tailor conversations about CMV by their own perception of a pregnant women’s risk. Some concerns were raised that pregnant families that were considered as ‘fragile’ or ‘overwhelmed’, and more information about CMV infection could cause upset.

“Parents get easily overwhelmed. especially when it’s (…) a new-born and (...) might be their first baby and (…) it can be quite an overwhelming experience anyway, and so we need to kind of... Minimize adding that you know at the extra worry onto it.” (P8, Community Midwife, 2.5 years)

Participants expressed concerns about how pregnant women would react to the message which discourages kissing children on the lips. CMV behaviour changes were described as associating *‘affection with infection’* which makes it uniquely difficult to promote such changes to pregnant women. This was particularly relevant to pregnant women who may be caring for younger children alongside their pregnancy.

“Yeah, because they just can’t bear the idea of tell me how to kiss my kid and then it’s actually, what happened is, it goes off down this whole social media conversation of whether you kiss your kid on the lips or not.” (Private Digital provider, 14 years experience)

Participants who perceived the film to provide practical solutions to these concerns and achievable modifications to behaviours in a non-frightening way, appeared likely to implement CMV in antenatal education using the intervention.

“The resistance I’ve heard about, was some people saying don’t tell me how to kiss my child, but in there, I like the way it said it was only a short time change and... And it’s just as good to kiss them on the forehead and a cuddle and that, so that that bit I liked a lot. If you sort of [look for] ideas and solutions, it’s not that difficult.” (P3, Midwife, 20 years experience)

In some cases, the short film was perceived to lack representation and cultural and socioeconomic diversity. This was considered an issue for antenatal educational resources in general too. Interventions to address existing inequalities in maternal care and the need for more effective and tailored care that traverses socioeconomic and cultural differences was seen to be imperative for successful implementation of any educational tool in pregnancy.

“Kissing on lips is a British thing... I believe in some culture you would never kiss a child on the lips because that is seen as sexual” (P2, Consultant Midwife, 27 years experience)

“I didn’t see any black babies... Black families. And I mean we aren’t just midwives, we aren’t just carers. We are also mothers as well. So maybe would have been nice to see more diverse imagery or videos of children or mothers there.” (Private organisation with interest in antenatal care, 5 years experience)

“Particularly in maternity, the wealth of resources for people whose first language isn’t English is tiny….we probably serve some of the most diverse population.” (Diversity research fellow lead, 1 yr of experience)

“Because for long, I’ve always felt that our voices weren’t necessarily included or had a prominent space in most decision making and procedures that ultimately impacts us, and arguably impacts us the most, since we are at higher proportionate rates dying in our pregnancy. We are more likely to have stillborn children, more likely to have emergency C sections.” (Organisation with interest in antenatal education, 5 years experience)

Some organisations recommended strategies to ensure more effective messaging and dissemination to a diverse audience, demonstrating the need for a wide approach to cognitive participation.

“Animation always works brilliantly and that also ensures that, I think for me from a diversity point of view that we are kind of reaching lots of different kinds of people, and that people relate to information.” (Private digital antenatal provider, 2 years)

#### Collective Action: Operational work, opportunities and barriers to implementation at an organisational level

Participants highlighted collective actions within local hospitals that could either support and facilitate successful implementation of a new practice or hinder it, leading to implementation failure. Information about CMV was perceived as fitting seamlessly alongside other educational topics at the initial ‘booking’ appointment, and therefore may make it easier for hospitals to potentially sustain the provision of this information (dependent upon the booking systems present at individual sites).^1^

“…so, it’s probably more at your booking appointment ‘cause you want them to sort of start getting that routine in as early as possible.” (P1 Midwife, Antenatal screening coordinator, 7 years experience)

“My concern is that I think the booking appointment is quite a chocker [busy] appointment as it already is. We know from some research we’ve done that there’s a certain percentage that women don’t retain because they’re given so much.” (Organisation with interest in antenatal education, 17 years experience)

Antenatal care providers recognised the importance of collectivising dissemination of the intervention through existing digital platforms within the hospital, in order to successfully integrate the intervention into routine care. These included showing the film on screens in antenatal waiting rooms, alongside other educational content, and existing social media platforms already utilised by the hospital.

“I think all the screens in all waiting rooms should have it. I don’t think that should be linked to only pregnant women. I think it should be on any screens. I think any TV that you have in hospital areas it should be promoted to each of these areas.” (P2, Consultant Midwife, 27 years experience)

“CMV is an important thing. But in the massive information that we have to do in that booking appointment, we’re fully aware that sometimes there’s important information gets lost because you’re bombarding people with lots of kind of thing, so actually having it up in a clinic is really useful because people can be sat there and watching it and it’s, you know, it’s relatively straightforward… So I think having it on a screen is definitely far, much more useful than a midwife trying to in a booking in appointment cover CMV.” (Private digital antennal provider, 2 years experience)

Midwives would then be reliant on wider services (such as Bounty, Best Beginnings, Wessex Healthier Together and Clevermed antenatal electronic notes) to signpost women to a trusted source to support their conversations and answer questions about CMV infection in pregnancy. These platforms may create collective opportunities to regularly update and integrating high-quality resources and provide more consistent information to pregnant women, overcoming variations in practice in different NHS hospital trusts. Midwives recognised these spaces as advantageous to the sharing of the CMV film as well as to the integration of the film into their routine practice. These external platforms include certain apps, websites, and baby information leaflet packs.

“I mean with COVID we’ve all seen a video, so whether there was a video where we could do- an hour’s educational video, and sort of say to women as we signpost them to look, this is a video. Please watch this.” (P10, Midwife, 13 years experience)

“Using our system helps midwives work more collaboratively, have more consistency with the information they’re giving, and less variations in the practice.” (Public antenatal provider, 7 years experience)

More experienced and senior members of staff were perceived as knowledgeable and as a useful resource to keep up to date with practice changes and their inclusion to assist with the integration of CMV as on-site ‘CMV champions’ were seen to be integral to success of integrating and sustaining a new practice.

“…Within the community setting and our [NHS organisation] we have quite a good, we’ve got a really good community matron and she is very good at like updating us and making sure that we’re... You know, uhm, we are adapting to changes that we need.” (P6, Community Midwife, 4 years experience)

“It really does feel like CMV is an issue where you need a sense of champions.” (Digital antenatal provider, 2 years experience)

NHS hospitals that had existing processes to approve or check new content, such as a Maternity Guideline Group or mechanisms to update and collect internal feedback were perceived to be more likely to implement interventions. This process could facilitate collectivised staff buy-in to the new intervention or process.

“The leaflets, videos, whatever are generally signed off by the Maternity Guideline Group… If you want anything to go on a guideline or on the app it goes to that group and we all have a look at it and decide yay or nay? And then, obviously, once it’s approved, it then gets uploaded.” (P13, Midwife, 7 years experience)

“If something is changing that gets circulated, sent out to staff, when people put in their comments and then it kind of goes through another governance check. And then they can officially be put either on our booklets, online, and websites.” (P9, Midwife, 5 years experience)

#### Reflexive monitoring: appraisal work Systemic-level Opportunities and Barriers

In addition to the NHS hospital trust facilitators and barriers, there was an acknowledgement by antenatal care providers of the wider systematic issues that play a role in the success or failure of implementation that would need reflexive monitoring. Participants highlighted a need for wider changes that would not only support implementation, but the sustainability of a new practice. This included: (1) national drivers and clearer guidelines for NHS hospital Trusts to consistently include antenatal education, (2) clear guidelines or policies relating to testing and screening for CMV, and (3) practices which consider issues of equality, diversity and inclusion (EDI).

Our participants felt that the lack of visibility of CMV infection within national policy leads to challenges relating its acceptance into practice, indicating the need for a “nationwide driver”. Without information about CMV being in the audit of important issues to discuss within antenatal education, the issue is not made a priority.

“You know our national requirements, where we are audited, etc. are going to be the priority…. CMV is important and we do want to put it somewhere, somehow, but without the push from above its hard to drive change.” (P12, Quality Improvement and Governance Midwife, 6 years experience)

“It has to be a change… it almost has to be like a nationwide one, a nationwide driver, to have that included so it could go into there.” (P9, Midwife, 5 years)

The importance of inclusion of the topic of CMV in the national agenda and endorsement from NHS bodies, such as the Royal College of Midwives (RCM), Royal College of Obstetricians and Gynaecologists (RCOG) or NHS England were reiterated by our participants.

“What we decide to promote on social media either has come because it’s like a national… Stoptober, all that kind of thing, or it’s that we’ve got particular agendas within the region, like COVID vaccinations.” (Public digital antenatal provider, 7 years experience)

“Well, the most important thing is to get it on the NHS website because nobody likes to link to anything that isn’t the NHS. We know that’s the trusted brand.” (Organisation with interest in antenatal education, 7 years experience)

The lack of national screening for CMV infection in pregnancy was perceived by midwives to be an important barrier to implementing information about CMV into antenatal education. This led them to feel exposed and anxious about discussing CMV infection with women, as they were not able to offer screening to tell them whether they had been exposed to CMV either previously, or had acute infection currently. Some midwives described that women from countries where screening for CMV was more normal practice were surprised to find out this is not the case in the UK.

“the bits that I find difficult… Is one lady who I had in particularly was from Greece originally really wanted CMV testing, but trying to explain why we don’t do it, I think those sort of conversations are quite tricky. So I just ended up referring her to the consultants and the consultants emailed me back and said I’ll just do the CMV testing.” (P7, Midwife, 7 years experience)

Midwives highlighted that when there were unclear pathways and barriers associated with requesting testing for CMV infection within their hospital site, they were unsure how to effectively integrate conversations about CMV infection into their practice. More junior staff felt worried about requesting a blood test that they do not normally request as part of routine practice.

“And it will probably just be if you are concerned talk to your midwife or something like, you know at the end it is difficult to identify if you have had it so we don’t test for it or something... because I feel like it would generate quite a lot of questions... uhm to explain either why we’re not testing for it or what they can do if they have concerns, anxieties where they should go to seek support?” (P11, Midwife, 7 years experience)

“But as a junior, a junior midwife would never do that because, because of the fear of being chastised for causing, you know expense to the unit” (P2, Consultant Midwife, 27 years experience)

Pregnant women’s requests for CMV testing might increase with more knowledge about CMV, and therefore would need reflexive monitoring during implementation. However, a participant that regularly discussed CMV infection and risk reduction measures during routine antenatal visits, had not found that this led to an increase in requests from pregnant women to test for CMV infection.

“No. So …since I’ve been doing it, which is probably pre 2016… we’ve had one woman that’s had a test… Far, far more far more for parvo and chickenpox.” (P3, Midwife, 20 years experience)

#### Implementation process findings

**Fig. 1:**
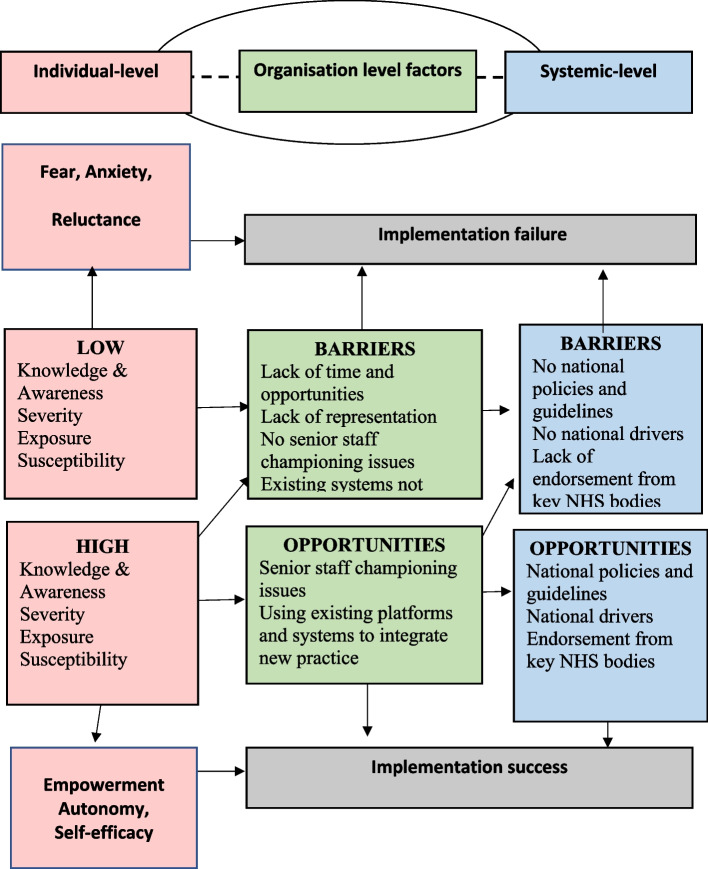
Predictive framework for implementation of antenatal education on CMV to reduce risk of infections in pregnancy

In addition to our qualitative findings, Fig. 1 provides a concept map of our implementation process findings. Through the perspectives of antenatal care providers, this model outlines how implementation could potentially result in either success or failure through individual-level factors, organisation level factors and wider systemic factors.

## Discussion

The findings of this study have both methodological and practice implications. Methodologically, there are implications surrounding the use of NPT to explore likely implementation success. And for practice, the study reports important perspectives from midwives and stakeholder organisations about the potential implementation of a CMV educational intervention into routine antenatal care. Our use of NPT is novel in the sense that most studies use it to reflect on the whole implementation process, whereas in our study we used NPT to identify the factors likely to cause issues before attempting implementation. We have found it to be a useful tool to organise service context and consider how best to normalise the intervention. In accordance with the NPT core constructs (coherence, cognitive participation, collective action, and reflexive monitoring), we identified several issues that would likely impact the normalisation of CMV education intervention within routine antenatal care.

Lack of knowledge and preparedness among midwives regarding CMV education and discussions with pregnant women is known to pose a significant barrier to the effective implementation of CMV education in routine antenatal care [11, 15, 17, 40]. Our study confirmed that midwives expressed confusion and a lack of shared coherence about CMV, but they perceived the short educational film as a valuable tool that can address these challenges. The use of this resource could increase midwives’ cognitive participation in navigating complex discussions on CMV, educating pregnant women within the wider context of communicating about risk in pregnancy. Any intervention highlighting the risks of CMV is also likely to increase the general sense of risk in pregnancy during antenatal care, which can engender a sense of ever-increasing surveillance of the otherwise healthy pregnant body, and impose an expectation that women will assume responsibility for managing risks which are difficult to quantify [41, 42]. The cause of this increased sense of risk may reflect wider cultural and biopolitical trends, beyond any single intervention. Where possible then, the implementation of CMV education should be sensitive to the effect it may have on service users. Antenatal healthcare professionals should have some scope to manage the emphasis of education, depending on the individual needs and circumstances of the pregnant woman. The integration and utilization of the intervention into routine practice is likely to need healthcare providers to consider where to place the resource in care pathways, when and where it should be shared, and by whom.

To address these challenges, there is a need for collective action and support from NHS Trusts to provide on-the-ground assistance and education for midwives. Even though CMV education may now be recommended according to NICE guidance, the success and efficiency of implementation should focus on how this guidance can be built from the “ground up”, where antenatal care providers have the opportunity to discuss, and tailor how to impart CMV education to their local population [43].

Mandatory training modules can improve midwives’ knowledge and confidence in discussing CMV [24]. This will ensure that antenatal care providers are better able to signpost women to the useful resources. This could be further improved by including CMV as part of checklists of topics of discussion at the first antenatal visit and digital platforms used for routine care or accessed directly by women themselves. The dissemination of digital CMV resources to women and families are crucial to support the successful integration of CMV education [44, 45], and there is a significant lack of existing information provision for CMV [46]. Tailoring guidance to fit each service’s internal platforms and assigning on-site champions should help to ensure the intervention is reflexively monitored and its usefulness evaluated, further supporting implementation [47]. This work could also be bolstered by a national steer from Royal Colleges to help to ensure that CMV is discussed as part of antenatal care, as recommended in the NICE antenatal care guidelines (NG201).

Education to midwives could also be strengthened with clearer guidance and monitoring on routine CMV screening and testing in routine practice. Midwives express concerns about pregnant women’s anxiety and the complexity of requesting CMV testing. Providing clearer guidance about testing pathways could address these concerns and facilitate early detection and intervention. Assuring midwives that routine discussions about CMV do not necessarily lead to increased testing may help alleviate their reservations about discussion of CMV with pregnant women.

By addressing these barriers at both the systemic and managerial levels, effective CMV education and discussions could be sustainably integrated into routine antenatal care, benefiting both midwives and pregnant women.

## Conclusion

By using NPT, we have been able to identify a range of barriers and facilitators that would affect CMV education becoming normalised or embedded within routine antenatal care. Barriers include a need for more knowledge, training, digital resource integration, and routine screening; facilitators include the willingness of midwives and organisational providers to share evidence-based CMV information with women. However, the issue of addressing midwives’ knowledge, its coherence and the extent of cognitive participation, only highlights part of the implementation challenge. More work is needed to provide a consistent approach for antenatal services to adopt these new practices. The study highlighted the need for collective changes to national and NHS organisational policies, accompanied by an understanding of each site’s digital systems and practice ‘norms’. Utilising an NPT framework in this context has helped in allowing us to explore local implementation factors [48]. However, our study also demonstrates that there is more scope to explore how organisations can work collectively when implementing to make changes to their practices, policies, and educational provision to implement the intervention into practice. It is not yet possible to state exactly where the resource needs to be used or what needs to change, as these are dependent on local factors. Many of the issues described by midwives as limiting them implementing CMV education in antenatal care stem from barriers emanating from existing organisational and macro societal structures [49]. Whilst focusing on the practice level we have been able to identify many structural barriers; there is an evident need for further work to identify the steps required for successful implementation. Although the blueprint for these steps may look similar (e.g., to identify where the resource should sit, who should be responsible, when it is imparted and by who), each step is likely to need localised interpretation and monitoring to fit the unique pathways and structures of specific antenatal care providers.

### Supplementary Information

Supplementary Material 1.

## Data Availability

The datasets used and/or analysed during the current study available from the corresponding author on reasonable request.

## Footnotes

1 In the UK health service facilities in this study, the first appointment is referred to as a ‘booking’ appointment which would typically take place before women are 10 weeks pregnant and involves some preliminary tests and questions.
