# Supplementary material for: Exploring the implementation of an educational film within antenatal care to reduce the risk of cytomegalovirus infection in pregnancy: A qualitative study
Source: BMC Pregnancy Childbirth. 2024 Aug 10;24:524. doi: 10.1186/s12884-024-06715-5 (PMC11316991; doi:10.1186/s12884-024-06715-5)
Supplement: Supplementary file 1 — Supplementary Material 1. [file 12884_2024_6715_MOESM1_ESM.docx]

Appendix 1: Detailed list of theme mapping process

| **Themes** | **Sub-themes** | **NPT component** | **NPT core construct** |
| --- | --- | --- | --- |
| CMV is not serious | Severity | Individual specification | Nature & attitude towards condition = Coherence |
| CMV does not cause harm | Severity | Individual specification | Nature & attitude towards condition = Coherence |
| CMV education not part of routine care now? | Severity | Internalization | Nature & attitude towards condition = Coherence |
| CMV transmission rates are low | Susceptibility | Communal specification | Nature & attitude towards condition = Coherence |
| 2nd time pregnant women more at risk | Susceptibility | Differentiation | Nature & attitude towards condition = Coherence |
| CMV is not common | Exposure | Individual specification | Nature & attitude towards condition = Coherence |
| CMV is not often seen | Exposure | Individual specification | Nature & attitude towards condition = Coherence |
|  |  |  |  |
| Initiates CMV conversations | Awareness & knowledge | Initiation | Motivation to engage with intervention = Cognitive participation |
| Increases awareness among staff & pregnant women | Awareness & knowledge | Enrolment | Motivation to engage with intervention = Cognitive participation |
| More confident to discuss CMV | Self-efficacy Autonomy & Empowerment | Enrolment | Motivation to engage with intervention = Cognitive participation |
| Pregnant women should take ownership | Self-efficacy Autonomy & Empowerment | Activation | Motivation to engage with intervention = Cognitive participation |
| My responsibility to educate pregnant women | Self-efficacy Autonomy & Empowerment | Legitimation | Motivation to engage with intervention = Cognitive participation |
| Messages are empowering | Self-efficacy Autonomy & Empowerment | Legitimation | Motivation to engage with intervention = Cognitive participation |
| Messages can scare women | Fear & anxiety | Activation | Motivation to engage with intervention = Cognitive participation |
| Messages are reassuring and comforting | Fear & anxiety | Legitimation | Motivation to engage with intervention = Cognitive participation |
| Women overwhelmed by antenatal education | Fear & anxiety | Activation | Motivation to engage with intervention = Cognitive participation |
| Concern for pregnant women welfare | Fear & anxiety | Activation | Motivation to engage with intervention = Cognitive participation |
| Kissing on the lips only relevant to some cultures | Representation | Enrolment | Motivation to engage with intervention = Cognitive participation |
| Black families need to be represented | Representation | Enrolment | Motivation to engage with intervention = Cognitive participation |
| Animations useful to show diversity | Representation | Legitimation | Motivation to engage with intervention = Cognitive participation |
| Intervention that address existing health inequalities | Representation | Legitimation | Motivation to engage with intervention = Cognitive participation |
|  |  |  |  |
| New resources signposted using existing resources | Integrating into existing NHS practices | Contextual Integration | Opportunities and Barriers at NHS Trust level = Collective action |
| Utilise booking appointment to signpost | Integrating into existing NHS practices | Contextual Integration | Opportunities and Barriers at NHS Trust level = Collective action |
| Show intervention in waiting rooms | Integrating into existing NHS practices | Contextual Integration | Opportunities and Barriers at NHS Trust level = Collective action |
| Social media to raise public awareness | Integrating into existing NHS practices | Relational Integration | Opportunities and Barriers at NHS Trust level = Collective action |
| Use of private antennal digital providers | Integrating into existing NHS practices | Contextual Integration | Opportunities and Barriers at NHS Trust level = Collective action |
| Lack of time to incorporate changes | Time & opportunities | Interactional Workability | Opportunities and Barriers at NHS Trust level = Collective action |
| Senior staff support changes & champion issue | Time & opportunities | Skill set Workability | Opportunities and Barriers at NHS Trust level = Collective action |
| Mechanisms to update and collect internal | Time & opportunities | Contextual Integration | Opportunities and Barriers at NHS Trust level = Collective action |
|  |  |  |  |
| Endorsement from recognised national bodies | National drivers and guidance | Systematization | Systemic level Barriers and Opportunities = Reflexive monitoring |
| Visibility of CMV within national policy and audit requirements | National drivers and guidance | Communal appraisal | Systemic level Barriers and Opportunities = Reflexive monitoring |
| Unclear pathways to request testing | Testing & screening policies | Communal appraisal | Systemic level Barriers and Opportunities = Reflexive monitoring |
| Costs associated with testing | Testing & screening policies | Reconfiguration | Systemic level Barriers and Opportunities = Reflexive monitoring |
| Lack of routine screening policies | Testing & screening policies | Systematization | Systemic level Barriers and Opportunities = Reflexive monitoring |
